# Preventative health assessments and indigenous people of Australia: a scoping review

**DOI:** 10.3389/fpubh.2023.1168568

**Published:** 2023-09-06

**Authors:** Kim Usher, Debra Jackson, Humayun Kabir, Rikki Jones, Joe Miller, Rachel Peake, Reakeeta Smallwood

**Affiliations:** ^1^School of Health, University of New England, Armidale, NSW, Australia; ^2^School of Nursing and Midwifery, University of Technology Sydney, Sydney, NSW, Australia; ^3^Faculty of Medicine and Health, School of Nursing, University of Sydney, Sydney, NSW, Australia; ^4^Department of Sociology, University of Dhaka, Dhaka, Bangladesh; ^5^Aboriginal Advisory Group Member, University of New England, Wollongong, NSW, Australia; ^6^Stroke Coordinator, Hunter New England Health, Newcastle, NSW, Australia

**Keywords:** indigenous, Australian, indigenous health assessments, preventive health, barriers, scoping review

## Abstract

Given that Indigenous populations globally are impacted by similar colonial global legacies, their health and other disaprities are usually worse than non-indigenous people. Indigenous peoples of Australia have been seriously impacted by colonial legacies and as a result, their health has negatively been affected. If Indigenous health and wellbeing are to be promoted within the existing Australian health services, a clear understanding of what preventive health means for Indigenous peoples is needed. The aim of this scoping review was to explore the available literature on the uptake/engagement in health assessments or health checks by Indigenous Australian peoples and to determine the enablers and barriers and of health assessment/check uptake/engagement. Specifically, we aimed to: investigate the available evidence reporting the uptake/engagement of health checks/assessments for Australian Indigenous; assess the quality of the available evidence on indigenous health checks/assessments; and identify the enablers or barriers affecting Indigenous persons’ engagement and access to health assessment/health checks. A systematic search of online databases (such as Cinhl, Scopus, ProQuest health and medicine, PubMed, informit, google scholar and google) identified 10 eligible publications on Indigenous preventive health assessments. Reflexive thematic analysis identified three major themes on preventive health assessments: (1) uptake/engagement; (2) benefits and limitations; and (3) enablers and barriers. Findings revealed that Indigenous peoples’ uptake and/or engagement in health assessments/check is a holistic concept varied by cultural factors, gender identity, geographical locations (living in regional and remote areas), and Indigenous clinical leadership/staff’s motivational capacity. Overall, the results indicate that there has been improving rates of uptake of health assessments by some sections of Indigenous communities. However, there is clearly room for improvement, both for aboriginal men and women and those living in regional and remote areas. In addition, barriers to uptake of health asessments were identified as length of time required for the assessment, intrusive or sensitive questions and shame, and lack of access to health services for some. Indigenous clinical leadership is needed to improve services and encourage Indigenous people to participate in routine health assessments.

## Introduction

Given that Indigenous populations globally are impacted by similar colonial global legacies, their health and other disaprities are usually worse than non-indigenous people (1). This is similar to many other Indigenous groups across the globe (2, 3). In Australia, Indigenous populations also have higher reported morbidity and mortality rates (4, 5) increased susceptibility to chronic disease (1, 5, 6), and lower rates of engagement and access to preventative health care (4, 5, 7) due to the many factors including the lack of access to appropriate services and racism (6, 7). Indigenous people in Australia represent approximately 3.3 percent of the total population. However, this rate differs significantly between States and Territories and urban and rural/remote locations varying between 1.8 percent in major cities to 32 percent in remote and very remote locations (8). As preventative health care is known to have a positive impact on the management of chronic conditions (6), it is important to understand Indigenous peoples’ access to these services and the enablers and barriers that affect access to available services.

Recent research suggests the rate of preventative health care being accessed has decreased during the recent pandemic (7), which is likely to have a negative impact on overall health of Indigenous people, especially those with chronic disease. Barriers to accessing preventative health care includes rurality, affordability, availability, lack of awareness of preventative health care services, and inappropriate services/resources (4, 5). Understanding the barriers and enablers of access to preventative health care by Indigenous people is important to help improve access to preventative health care services and to improve the overall health of communities. Hence, a scoping review is timely to explore the available literature related to Indigenous persons’ health-seeking behaviours regarding preventative health care.

Primary, secondary and tertiary prevention is defined as the following: Primary prevention focuses prevention of disease using health promotion strategies and interventions to target at risk populations; Secondary prevention focuses on intervention such as early detection of disease through screening and interventions; Tertiary prevention focuses on reducing the impact of an existing disease (9). For the purpose of this scoping review we will focus on health assessment or health checks which are used as both a primary and secondary prevention tool (10).

Health checks/assessments were identified in the Indigenous chronic disease package as preventative measure for chronic disease (1, 5) and as a key performance indictor of health by the National Indigenous Reform Agreement (5). Health assessment was first introduced into Medical Benefits Scheme (MBS) for Indigenous and Torres Strait Islander people aged over 55 years in 1999 and for those aged between 15 and 54 in 2004 (11). The main item number for Indigenous health assessment in Australia is MBS 715, which usage rate, according to Australian Bureau of Statistics, increased from 11% in 2010–11 to 29% in 2016–17 (12). This demonstrates an increase in engagement in the health assessment/check for Indigenous people, but indicates that more needs to be done to increase engagement and access to have an impact on health outcomes and chronic disease for Indigenous Australians. However, it is important to recognise that access to preventive health varies across Australia with people living in regional, rural and remote locations having less access to these services (13). Given the importance of this information to the future development of the preventive health check strategy for Indigenous people in Australia, we aimed to summarize the exisiting evidence using a standardized scoping review methodology.

The aim of this scoping review was to explore the available literature on uptake/engagement in health assessment or health check, for Indigenous Australian peoples and to determine the barriers and enablers of health assessment/check uptake/engagement. Specifically, we aimed to map the (1) evidence reporting the uptake/engagement of health checks/assessments for Australian Indigenous; and, (2) the enablers or barriers affecting Indigenous persons’ engagement and access to health assessment/health checks.

## Methods

### Design

A scoping review methodology was selected as it was deemed the most appropriate method to explore and examine the available evidence in this specified field, and to allow the research team to provide a scope of what is reported in the literature around a particular concept, to identify gaps in the literature, and highlights areas of future research (14, 15). The eligibility criteria for inclusion and exclusion of literature in this review were determined using the PCo (Population and Context).

Inclusion Criteria

1. Studies were included if indigenous perspectives were found for a study population with uptake/barriers to healthcare facilities.
2. Indigenous peoples worldwide
3. Published in English
4. Original research including qualitative, quantitative and mixed methods. Grey literature includes Google Scholar
5. Full text available

Exclusion Criteria

1. Literature reviews (relevant articles from these included), commentaries, editorials, book reviews, letters to the editor, or where the full text was not available.
2. Non-English publications

### Search terms

Indigenous OR First Nation* OR Aborig* OR Torres Strait Islander AND Health Assessment OR Health Check

### Search strategy

The search included a comprehensive strategy to identify the available literature pertaining to Indigenous health assessment/health checks uptake using the search terms. One reviewer performed and conduct the initial search of evidence to determine key terms and develop the search string. A health librarian was consulted to ensure the databases and search string would produce the desired results. The search was pilot tested in one database (selected by the research team) to ensure the search strategy was robust enough to capture the required evidence, before the search strategy was finalised. The search was then conducted by two researchers across the following databases Cinhl, Scopus, ProQuest health and medicine, PubMed, informit, google scholar and google. For each database the relevant papers were identified and the reference, title, abstract and keywords were exported as .Ris file into EndNote and duplicates removed. The remaining results were exported to Covidence, where two reviewers undertook title and abstract screening followed by full text screening. The reviewers meet to discuss any conflicting decisions, if the two reviewers were unable to make a final decision a third reviewer was consulted and final decision made. The PRISMA flow chart (16) was used to report results of the screening process. It is worth mentioning that given critical appraisal and risk of bias is not required for scoping reviews (17), this was not included in the manuscript.

### Data extraction and analysis

One reviewer extracted data from the evidence included in the review using the standardized Joanna Briggs Institute data extraction and checked by a second reviewer. The data extraction tool gathers specific information on population, context, culture, geographical location, study methods, the phenomena of interest relevant to the review objectives, and source type. Disagreements between the two reviewers were resolved through discussion, or by a third reviewer. A reflexive thematic analysis was undertaken to extract findings under common themes. Following the six steps narrated by Braun and Clarke (18), which was commonly used in the previous health research (19, 20), reflexive thematic analysis was done. First, two authors familiarised with the data of the finally included studies, followed by deep immersion with the data by reading and re-reading. Second, initial coding was then generated. Third, the codes were used to form initial themes. Fourth, the collapsing and refining the codes and themes. Fifth, identifying the story in support of the themes. Finally, a review of the themes and subthemes was conducted to check whether they reflected the meaning of the coded extracts and data set appropriately. The findings were reviewed by the research team.The findings and illustration of findings are available in the Supplementary file.

## Results

**Figure 1:**
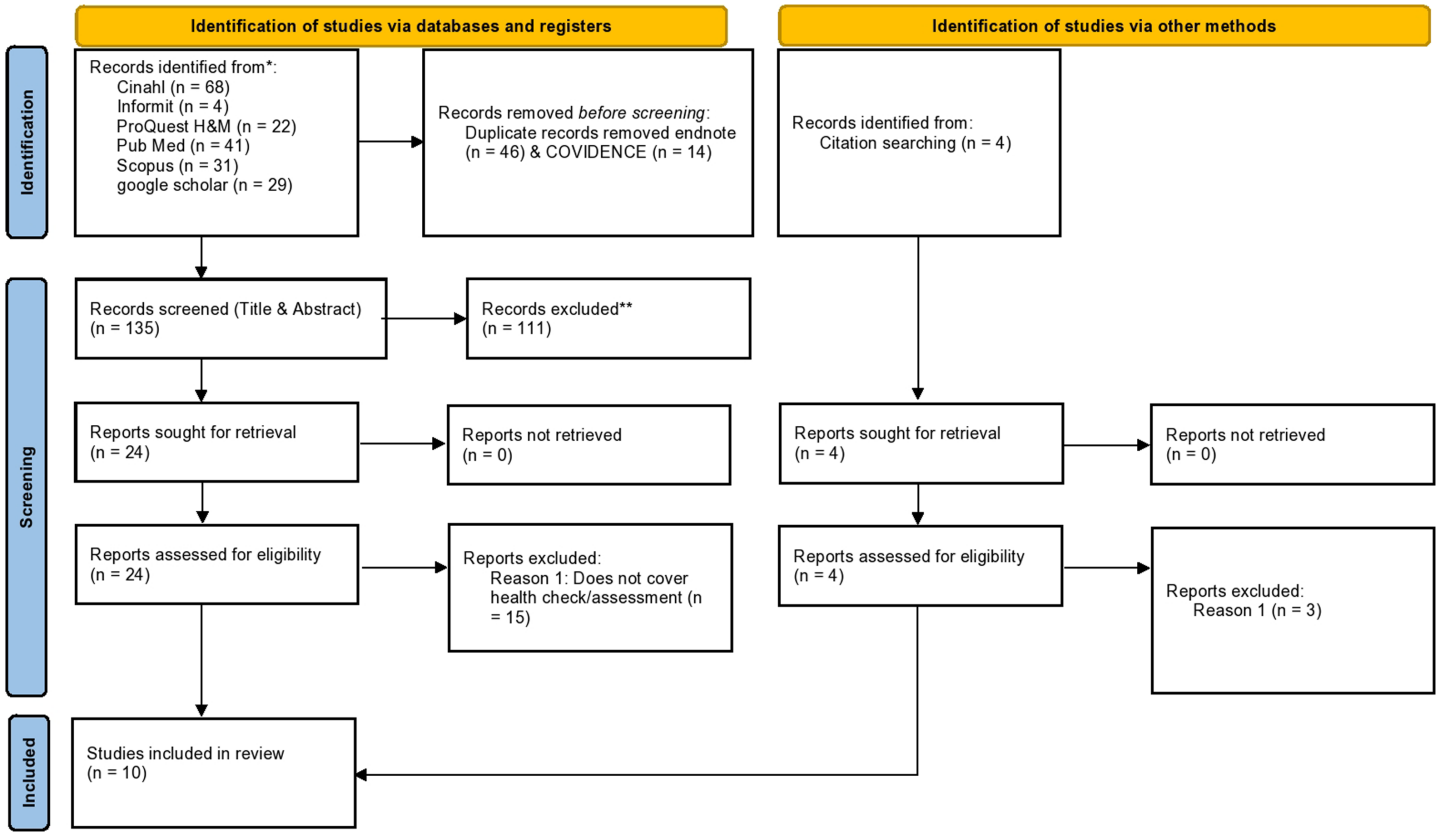
PRISMA flow chart.

The search identified 195 sources of literature, 60 duplicates were removed, leaving 135 for title and abstract screening. Of these 24 were retrieved for full-text screening, of which 9 meet the inclusion criteria. Reference lists of these 9 papers were searched and a further 4 were identified for title and abstract screening of which only 1 meet the inclusion criteria (*n* = 10; see Figure 1).

A quality assessment/risk of bias was undertaken of the included studies using the Mixed Methods Assessment Tool (MMAT) (21). All 10 studies were rated as medium to high quality, Table 1 presents the full results of the quality appraisal.

**Table 1:** Quality appraisal MMAT.

Methodological quality of the included quantitative (descriptive) studies using MMAT (yes = 1, no = 0)									
Study	Clear research objectives/questions	Data addressing research objectives/questions	Relevancy of sampling strategy	Sample representativeness of target population	Appropriateness of measurement	Low risk of nonresponse bias	Appropriateness of analysis to answer research question	Total points	Ratings (6–7 = high quality, 4–5 = medium, >4 = low quality)
Reid et al. (20)	1	1	1	0	0	0	1	4	Medium
Methodological quality of the included qualitative studies using MMAT (yes = 1, no = 0)									
Study	Clear research objectives/questions	Data addressing research objectives/questions	Appropriate approach to answer research question	Adequate data collection methods to address research question	Findings adequately derived from data/adequate data analysis	Interpretation of results sufficiently verified by data	Coherence between data sources, collection, analysis, and interpretation	Total points	Ratings (6–7 = high quality, 4–5 = medium, >4 = low quality)
Jennings et al. (19)	1	1	1	1	1	1	1	7	High quality
Schütze et al. (3)	1	1	1	1	1	1	1	7	High quality
Spurling et al. (2)	1	1	1	1	1	1	1	7	High quality
Bailie et al. (4)	1	1	0	1	1	0	1	5	Medium
Methodological quality of the included quantitative (non-randomized) studies using MMAT (yes = 1, no = 0)									
Study	Clear research objectives/questions	Data addressing research objectives/questions	Participants representativeness	Measurements appropriate	Complete outcome data	Confounders accounted in design and analysis	Conducting intended intervention	Total points	Ratings (6–7 = high quality, 4–5 = medium, >4 = low quality)
Butler et al. (14)	1	1	1	1	1	1	1	7	High quality
Dutton et al. (15)	1	1	1	1	1	1	1	7	High quality
McAullay et al. (16)	1	1	1	1	1	1	1	7	High quality
Panarett et al. (17)	1	1	1	1	1	1	1	7	High quality
Robertson et al. (18)	1	1	1	1	1	1	1	7	High quality

The characteristics of the studies included in this review are presented in Table 2. Of the 10 studies, seven studies collected quantitative data (*n* = 7) and three studies collected qualitative data (*n* = 3). All studies discussed Indigenous engagement with in regard to preventative health care, however few studies presented the view of Indigenous community members.

**Table 2:** Literature characteristics.

Citation	Aims/objectives	Study Design and methodology	Sampling	Analysis methods	Overall results	Country
Bailie et al. (4)	To describe patterns of uptake of Indigenous-specific health assessments and associated follow-up items, and examine the barriers and enablers to delivery and billing of follow-up over the first 3 years of implementation of the Indigenous Chronic Disease Package (ICDP)	Quantitative data- The SSE was a formative evaluation covering 24 urban, regional and remote locations in all Australian states and territories. Data were collected, analysed and reported in 6-monthly intervals over five evaluation cycles between 2010 and 2012 Quantitative data from-Focus groups, in-depth interviews and discussions with key informants.	Purposive Of the 581 individual interviews done through the Sentinel Sites Evaluation (SSE), 63 contained specific information about the follow-up of health assessments Of the 58 group interviews, 31 contained information relevant to this study, which included 103 participants Of the 72 community focus groups, 69 provided data on access to services Qualitative data on barriers and enablers to delivery of and billing for follow-up were obtained from individual and group interviews with a range of key informants from Aboriginal Health Services (from SSE)	Analysis of SSE data using a socioecological framework Thematic analysis Used an iterative approach to categorise these themes	Aggregated data show a general improvement in uptake of health assessments and follow-up items after the baseline period Barriers and enablers to delivery and billing of follow-up care using a socioecological framework were identified at five levels of influence: patient, interpersonal, health service, community and policy. Negative past experiences affected patients’ willingness to attend follow-up appointments. Health service providers felt that short consultation times meant they had limited opportunity to explain reasons for referral for follow-up care to patients. This was related in part to shortage of service providers, including GPs, allied health professionals, Aboriginal Health Workers (AHWs) and practice nurses Barriers related to Indigenous social and economic disadvantage included poor availability of transport to attend follow-up appointments and high or unpredictable cost of allied health services.	Australia
Butler et al. (14)	To quantify claims for the Aboriginal and Torres Strait Islander health check (MBS item 715) in a 2-year period among Aboriginal and Torres Strait Islander adults from the general population of New South Wales, Australia, in relation to sociodemographic and health characteristics, including prior CVD and CVD risk factors	Quantitative Survey questionnaire Self-reported baseline questionnaire	Random sampling The study involved 1753 Aboriginal and Torres Strait Islander adults	Frequencies and proportions were calculated for the sample according to participant characteristics, for the total sample and by claim for a health check Logistic regression was used to estimate odds ratios (ORs) and 95% confidence intervals (95%CI) for receiving a health check in relation to participant Characteristics The significance of the addition of an explanatory variable to the model was determined using the Wald joint test of significance. Analyses were undertaken using Stata 14.1	Approximately one-third of participants received a Medicare-funded health check over a 2-year period in this large population-based study of Aboriginal and Torres Strait Islander adults living in NSW, those who were disadvantaged, lived remotely, had CVD risk factors or established CVD, and had poorer self-rated health were the most likely to receive a health check. Overall, 32% of participants had received at least one health check in the 2-year period from 1 January 2014 to 31 December 2015 Use of GP services and poorer self-rated health remained strongly associated with receiving a health check Most participants (91%) made at least three GP visits per year in the follow-up period, with 45% making more than 10 visits per year; 2% had no record of an MBS claim for a GP service.	Australia, NSW
Dutton et al. (15)	This study aimed to document (1) The number of each type (older person, adult, child) of AHA performed at the OAMS in 2011 and 2012; (2) The risk factors and new morbidities identified (3) The initial actions, management and 6-month follow up of any abnormalities detected	Quantitative Retrospective data extraction clinical records (2 year study)	−1,169 AHAs were performed 41% child, 53% adult and 6% older person AHAs	Descriptive statistical analysis was performed using SPSS software (version 21).	1,169 AHAs were performed: 52% (612) in 2011 and 48% (557) in 2012 (Table 1). Of these, 148 had two AHAs, none had more. Twenty-six per cent of the Orange Aboriginal population received an AHA in 2012 The most common risk factors overall were being overweight and smoking For all cases that were overweight and for all participants who currently smoked and intended to quit, only 37 and 60%, respectively, received an intervention. Identification of skin and ear problems and poor dentition were similar. The OAMS more commonly identified hypertension (18% adults) compared with the Inala adult AHAs (12%). The OAMS changed information management systems in December 2011 and there may have been under-reporting of risk factors and intervention in the earlier period	Australia Orange
Jennings et al. (19)	The study sought to identify barriers and enablers to undertaking health checks in an urban Aboriginal Medical Service	Qualitative Semi-structured interviews	Purposive Of 30 clinical employees at the Aboriginal and Torres Strait Islander medical service (AMS), 25 staff (10 Aboriginal Health Workers (AHWs), 8 nurses and 7 doctors) participated in 20 interviews (five paired). Eight AHWs and three nurses identified as Aboriginal, and two AHWs identified as Torres Strait Islander. Three participants were male: one AHW and two doctors.	An inductive approach content analysis was used to identify patterns and themes in the Data NVivo 9 software was used to assist data management and analysis. All Aboriginal and Torres Strait Islander staff but one, explicitly and without prompting, identified community health promotion and outreach as important enablers to improve HC uptake	Data analysis revealed that successful completion of HCs was contingent upon several interconnected components, including the client attending the AMS and consenting to the HC, and staff initiating and completing it. Barriers and potential enablers were identified at each of these stages, in addition to overarching systems within the clinics. The AMS lacked a service-wide approach for conducting health checks (HCs), with different systems between clinics, and different systems recounted by staff within clinics Maintaining client-centeredness was a concern for many staff who identified competing priorities in clients’ sometimes stressful lives. The study provided important insights into the barriers (e.g., inadequate practice systems and a lack of confidence for some staff in HC initiation and undertaking lifestyle brief intervention, socio-culturally sensitive health check content, and a lack of community engagement with HCs specifically, and preventative health care in general.) and enablers of increasing HC uptake.	Australia; Brisbane
McAullay et al. (16)	The primary objective of this study was to determine whether participation in the ABCD programme was associated with improved care and outcomes for Indigenous children. A secondary objective was to assess if quality differed by geographic location.	Quantitative medical records audit Data were collected from 59 Australian primary health-care centres providing services to Indigenous people and participating in the programme 6-year study period (2008–2013) (February 2008 and December 2013)	Random sample 30 records from each clinic There were 2,360 individual file audits conducted in the 59 centres during the period February 2008 to December 2013. Only four were non-remote centres (323 individual file audits)	Crude and adjusted logistic generalised estimating equation models were used to examine the effect of year of audit on the delivery of care. Odds ratios (ORs) and 95% confidence intervals (95% CI) were calculated to compare the outcomes from 2008 to other subsequent years and to assess the time trend. Data analyses were conducted using STATA 13.1	Over the study period, the percentage of children included in recall systems significantly improved from 84% (*n* = 357) in 2011 to 95% (*n* = 415) in 2013 (OR 2.44, 95% CI 1.44–4.11) Complete data were available for all items except checks on parent–child interaction, skin and oral health, which were available from 2011 to 2013 only. Weight checks remained consistently high (96–98%) and haemoglobin checks remained low (52–66%) from 2008 to 2013. All other child health check items showed statistically significant improvements over time (skin, oral, ears, hearing, development, interaction) (Table 2). Hearing assessment improved the most, from 52% (*n* = 105) in 2008 to 89% (*n* = 378) in 2013 (OR 2.17, 95% CI 1.60–2.94). Skin checks improved from 73% (*n* = 309)	Australia; SSA, NT, WA
Panaretto et al. (17)	To examine improvements in the delivery of clinical care against key performance indicators.	Quantitative Longitudinal time point data (database- QAIHC) Indigenous and non-indigenous pts. presenting to a Queensland Aboriginal and Islander Community Controlled Health Service Data collection- June 2010 to February 2012	Convenience The study data have been collated from data extracted by the QAIHC Core Indicator report in the Pen CAT tool. The data collection thus represents a ‘live’ whole of the service patient snapshot.	Descriptive statistics usin SPSS v19 Proportions and 95% CIs or medians and IQRs using SPSS V.19.	Aboriginal and Torres strait islander Attendance at clinics increased from 273,692,010 to 55,441 in 2012 The aggregated performance of participating services for health assessment increased over time. In October 2011, 8,697 (44.1%—43.4, 44.8) of the regular patients had a current health assessment.	Australia Queensland
Reid et al. (20)	To integrate cultural considerations and developmental screening into a First Nations child health check	Quantitative (Questionnaire, descriptive statistics) A short questionnaire survey via phone was conducted	Convenience Participant’s presented to GP clinical and were eligible were entered into REDCap database (developed) A total of 118 children participated in the Share and Care Check between June 2019 and February 2020. Fifty-five caregivers consented for their child’s data to be used for research purposes, and 28 caregivers consented and participated in a short feedback questionnaire.	Descriptive statistics were reported as means and standard deviations for normally distributed continuous data, or medians and interquartile ranges for non-normally distributed data. Normality was assessed using a Shapiro–Wilk test. Categorical variables were reported as frequencies and percentages.	The current study provides: (1) preliminary outcomes documenting cultural connections and developmental needs; and (2) feedback from caregivers regarding their experience of the Share and Care Check. theme 2 reports findings related to this lit review All caregivers reported the Share and Care Check was culturally appropriate, and the majority also reported that it was helpful (n = 23; 85.2%). A key positive feature noted by caregivers (n = 11; 40.7%) was the comprehensive nature of the health check. However, four caregivers (14%) reported that the health check took too long.	Australia, Queensland Gidgee
Robertson et al. (18)	The study aimed to assess the impact of the COVID-19 pandemic on First Nations people health assessments using an interrupted time series model.	Quantitative (data extracted from Australian MBS database) MBS item numbers included 715 (face-to-face health assessments), and 92,004 and 92,016 (temporary COVID-19 telehealth services).	-Convenience	Additive triple exponential smoothing (TES) is a forecasting method used to model and predict observations in a time series (health assessments) Percentage differences between observed and predicted health assessments between January and June 2020 were calculated with 95% CI Observed values falling outside the 95% CI of the model’s prediction were considered statistically significant (*p* < 0.05).	There was no significant difference between observed and predicted First Nations people health assessments in January, February, and June 2020. However, we found a statistically significant decrease in health assessments in March (16.5%), April (23.1%), and May 2020 (17.2%). The proportion of total health assessments delivered via telehealth was 0.5, 23.6, 17.6, and 10.0% for March, April, May, and June 2020, respectively Telehealth health assessments did not entirely mitigate the reduction in face-to-face health assessments for First Nations people during the first wave of the COVID-19 pandemic	Australia
Schütze et al. (3)	This study explores some of the reasons why the uptake of Health Assessment for Aboriginal and Torres Strait Islander People remains low in some metropolitan general practices.	Qualitative Semi-structured interviews were conducted	Purposive In total, 31 out of a possible of 44 participants agreed to take part in the study (eight out of eight GPs, two of four nurses, one of one allied health professional, four of six practice managers, 16 of 25 receptionists).	Interviews were transcribed verbatim Thematic analysis was performed in Nvivo version 9.2. Authors reviewed the coding of five interviews to identify differing or additional insights or meanings, which then informed the subsequent analysis.	This study confirmed previously described barriers to MBS-715 uptake in general practice, including low rates of Indigenous status identification and a lack of awareness of MBS-715. Additional barriers found in this study were avoidance of billing health assessments	Australia
Spurling et al. (2)	This research aimed to identify the priority health issues of the Inala Aboriginal and Torres Strait Islander community	Qualitative The authors situated this research in the transformative paradigm Conducted face-to-face, semi-structured interviews	Purposive Twelve men and nine women took part in the interviews	Transcribed interview data were uploaded to NVivo 9 Thematic analysis was performed	3 central themes- (1) complex, interrelated, intergenerational nature of health involving social, cultural and environmental determinants of health (SCEDH); (2) ambivalence about HAs; and (3) community strength. Theme 2 reported findings related to this literature review. Most key informants had had a health assessment (HA) with only two saying they had never had one. Key informants’ experience of Aboriginal and Torres Strait Islander HAs were mixed, as four key informants gave unqualified support for the capacity of HAs to detect medical problems early. Participants’ responses suggested that their view of health and the social world was not adequately covered by HAs, which measured health in a compartmentalised, disease-focussed way.	Australia

Data analysis revealed three main themes which address the aims and objectives of the scoping review: (1) uptake/engagement; (2) benefits and limitations; and, (3) enablers and barriers. Table 3 presents a summary of themes reported by each study.

**Table 3:** Summary themes reported.

	Theme		
Citation	Uptake/engagement	Benefits and limitations	Enablers and Barriers
Bailie et al. (4)	X		
Butler et al. (14)	X		
Dutton et al. (15)	X	X	
Jennings et al. (19)		X	X
McAullay et al. (16)	X		
Panaretto et al. (17)	X		
Reid et al. (20)		X	X
Robertson et al. (18)	X		
Schütze et al. (3)		X	X
Spurling et al. (21)		X	

### Theme 1: uptake/engagement

Six of the 10 studies (1, 22–26) reported on indigenous people’s uptake of health assessments. The findings from Bailie et al. (1) and Panaretto et al. (25) indicate a general improvement/increase in the uptake of health assessments and attendance at clinics. The study conducted by Butler et al. (22) showed that approximately one-third (32%) of Aboriginal and Torres Strait Islander adults living in NSW received a Medicare-funded health check over a 2-year period. Besides the adults, another study observed an increase in indigenous child health checks recorded in medical records (24). One study (23) reported on engagement levels during 2011–2012, 1,169 health checks completed in Orange. However, there are also contradictory findings explored in a research conducted by Robertson et al. (26) which demonstrated statistically significant reductions in total First Nations people’s health assessments during the early wave of COVID-19 (March, April, and May 2020).

There are several predictors which played an important role in the Indigenous peoples’ uptake of health assessments. Research conducted by Butler et al. (22) found that women had more health check compared to men. The study also pointed out that health check varies depending on residency or locality (inner regional vs. outer regional). For example, the overall health check among the inner regional residents (33.3%) was higher than the outer regional residents (4.7%).

### Theme 2: benefits and limitations

Four studies (5, 23, 27, 28) have emphasised the early identification of chronic diseases and health risk factors, which can be done by timely health checks. The early identification of diseases protects the patient from further health complexities (5). The Indigenous community-targeted health design or project (such as the ‘Share and Care Check’) was found culturally appropriate to attract the Indigenous peoples for health checks in their childhood (28). The best part of ‘Share and Care Check’ is its comprehensive nature of health check. More benefits of health assessments have been noted in the existing research. Dutton et al. (23) explored that the primary benefit was identifying the common health risk factors which include overweight (41%) and smoking (26%). The second benefit was related to receiving advice from the health professional, vaccination, and referral. The primary identification of health problems among the patients opens the door for further investigation when required. For example, Dutton et al.’s (23) study showed that overall 41% of cases received advice; 27% were prescribed new medication; 13% were vaccinated; 41% had at least one blood test ordered and 32% had further investigation; 70% were given at least one referral, most commonly to a dentist; and 42% were advised to return for a review. Despite the higher rate of referral, it is worth noting that there is tendency of not completing the recommended dental care treatment among the Aboriginal clients (29). It is quite unknown why they are reluctant to uptake health workers’ referral for care. Time and costs associated with dental care could be an important reason of why Aboriginals are less keen to uptake medical care. Differences between medical care and dental care conditions that operate at a clinic or community level may affect uptake of dental care. This needs to be practically addressed. In addition to overweight and smoking, several new health problems (such as skin, ear, and dental problems) were also discovered during extended diagnosis. Other than the treatment-related benefits, another study [conducted by Jennings et al. (27)] focused on the financial benefits to clients, including subsidised medications and allied health consultations.

Spurling et al. (30) disclosed several limitations of health assessments. These limitations should be considered as obstacles for Indigenous peoples in accessing health services. One of the limitations is feeling shame to go to the doctor, which was evident from the following statements reported by Spurling et al. (30).

*“I think the health checks are really important for Aboriginal and Torres Strait Islander people because some people ….feel shame to go to the doctor, and if they leave it too long there could be a problem building in their body [Liam]”* (p. 551).

Sometimes medical professionals do not go to the in-depth level or to the heart of peoples’ health problems. This was considered a significant limitation in the study of Spurling et al. (30). The following statement would illustrate this clearly-

*“I’m not sure whether it paints a really honest picture of exactly where my health’s at. I think that [it] probably can go a bit more in depth [Edward]”* (p. 551).

Participants also mentioned that they felt identity crisis and/or their social world and health were not properly addressed by the existing health assessment procedures, which are mainly disease-focused. For example-

*“I don’t see how a doctor is going to solve an identity crisis. It’s a social thing… [Bradley]”* (p. 551).

### Theme 3: enablers and barriers

Three studies reported findings on this theme (5, 27, 28). Jennings et al. (27) proposed how to encourage Indigenous people attend regular health checks. Most felt that current community health promotion activities were inadequate, and the difficulties reaching an often-transient urban population were raised. Therefore, health promotion at the community level should be considered an important enabler to improve HC uptake. Indigenous community engagement alongside the direct promotion of the HC is necessary to make sure that more Indigenous people become interested in regular health checks. The study discovered that the existing community health activities were inadequate and hard to avail. The following statement of a participant mentioned in the study conducted by Jennings et al. (27) would make it clear-

*“You know, we need to advertise it …. we need client[s] to approach us and …. request it …. both parties have to agree and … like want it from the heart. We need to educate our people more. Tell them about the health check, the importance of health check. We need posters and pamphlets or whatever we can to provide that information to our clients, ‘cause I look at here … it’s like 20 years back [than at home] … like in the health knowledge of the community, like in the conscious[ness].’ (AHW H13)”* (p. 154).

There were several studies (5, 27, 28) who reported on the potential barriers to health checks. Firstly, Reid et al. (28) noted that the health check took too long. When health check procedures take a long time, Indigenous people may be less likely to seek this preventive opportunity. Additionally, Schütze et al. (5) found that GP services are much too time-consuming. In addition, due to the lack of strong local leadership and good communication, the development of clinic-specific systems is impossible to embed the HCs as a routine practice within busy workplace settings. By quoting a statement of a doctor, the study conducted by Jennings et al. (27) illustrated-

*“…it needs like a practice manager who’s there to make sure it’s functioning and without that it’s quite ad hoc …. and so doing something extra like a health check just becomes sort of an extra burden rather than a routine practice… (Dr H18)”* (p. 152).

The above statement can be seen as a reflection of the lack of services support for completion of health assessments, which is an obvious barrier to a routine health check. Medical professionals suggested important indicators (such as encouraging clinical leadership and positive attitudes with audit and feedback of health checks) to uplift motivation toward health checks which eventually elucidates the barriers to health checks (27).

The study by Jennings et al. (27) also noted disorganised management within the hospital/clinic settings. The following statement of a nurse clearly expressed the concerns about the difficulties of health check-

*“‘.… that no one’s got together and we don’t have a system’ (RN H20)”’* (27, p. 152).

In addition, another potential barrier is related to the business of the clinics and the time needed to complete health checks.

*“‘.… it’s like the size of War and Peace!’ (AHW H16A)”’* (27, p. 152).

*“‘.… with the Indigenous people.… you don’t keep them for a long time … otherwise they’ll just get up and go out.… (RN H01-Aboriginal) ’”* (27, pp. 152–153).

As a result of the above-mentioned barriers, it is important for health services to organise regular annual health assessment appointments for Indigenous people.

*“.… in order to get a health check here you have to have an appointment, so …. that’s one of the biggest barriers … you know ‘oh, come back next week for a health check, yeah?’ no, they’re not going to come back … they got what they need now … we really need opportunistic health checks … that’s what we need … (AHW H15)”* (27, p. 153).

Asking questions about lifestyle factors, particularly alcohol and smoking, but also the social history, including current home environment and overcrowding as a part of health check often deter Indigenous people from attending regular health assessment/checks services. This is not suoprising given the colonial history of child removal and other examples of colonoial interventions. Therefore, they consider health checks as difficult, sensitive, or invasive which can be illustrated by mentioning the statements used in the study conducted by Jennings et al. (27, pp. 153–154)-

*“I felt like it was [Department of Communities] you know, the department, asking some of those questions, ‘how many people living in your house?’.… that’s not too bad, it’s starting to get a little bit invasive but, ‘does the mother drink, does the father drink?’, ‘how much do they drink?’.… What’re we trying to achieve? We know we’re gonna get social issues with a lot of these kids. So once you find out that Dad drinks every, whatever, or they’re all smoking in the house, then what do you do? Again it comes back to capacity around implementing that information … (RN H07 Aboriginal)”*

*“.… I think the hardest thing about the health check probably for both parties would be the lifestyle stuff, because that’s the most personal …’* (Dr H18).”

## Discussion

The review reveals strong uptake of health assessments with some sections of Indigenous communities. However, there is clearly room for improvement, particularly with men and those living in regional and remote areas. Butler et al.’s (22) work showed gender disparity with more women than men undergoing health assessment. This is likely because women often attend health services more than men (21), particularly between the ages of 15 and 44 years, partially because of reproductive health issues (31), and also because women are often responsible for taking children and others in their care to doctors appointments. There is a need to increase Indigenous men’s engagement with regular health assessments, and in developing strategies for this to occur, it will be important for health services to engage in authentic and effective collaboration with Indigenous men to develop enhanced understandings of how services could be better configured to improve male participation. Strategies aimed at increasing uptake should consider cultural factors identified in this review such as potential for feelings of shame associated with the current questions included in health checks (30), and consider the need for an individualised approach to health assessment, with some evidence suggesting that assessments may be perceived by some Indigenous men as being superficial or lacking in adequate depth (30). There was also evidence that some Indigenous people felt the health assessment was too time consuming and this also affected uptake (28) as well as causing people to leave the clinic or hospital before the assessment is completed (27). Jennings et al. (27) also suggested a disorganised system in some disarray that was poorly prepared to meet the needs of community-dwelling Indigenous people. In light of these findings there is a need for closer collaboration with Indigenous communities and services, with a view to increasing efficiency of services, so that clients are not being left with a sense that their time is not being optimally respected and more likely to remain in the clinic until the assessment is completed.

There is clearly inequity in uptake of health assessments between inner and outer rural dwelling Indigenous people (22), and this inequity is mirrored in the broader population with outer rural dwelling people having less access to health services generally and associated poorer health outcomes (32). This disparity is often associated with distance and also with reduced health workforce availability. However, our review of the literature also suggests that urban-based services experience challenges associated with transient populations, and that Indigenous people may feel that community health services are inadequate to meet health needs, even in urban settings (27). Again, there is a need for enhanced and stronger collaboration between health services and Indigenous communities and specialist Indigenous-led services such as Aboriginal Community Controlled Organisations (ACCHOs) to work together to build stronger partnerships to enhance participation in health assessments. Furthermore, Jennings et al. (27) also highlighted the lack of clinical leadership in this important area, and that this lack of leadership resulted in services that lacked the motivation to undertake routine health assessments, or to work to streamline and improve service users experiences of health assessments. Therefore, we recommend that Indigenous clinical leadership is needed to improve services and to inspire and motivate Indigenous people and clinical staff to better participate in routine health assessments. In addition, we argue that strong Indigenous clinical leadership will provide an important clinical and cultural link between key stakeholders and this will also likely go some way towards improving community participation. It is therefore imperative that further research be conducted to better understand the reality of Indigenous peoples’ preventive health attendance and access to services in Australia.

Of concern was the lack of literature that is from the perspective and voice of Indigenous people. The lack of Indigenous perspective means that the current evidence lacks an important position that must not be ignored.

### Limitations

As with all reviews, there are some limitations to acknowledge in this review. Firstly, reports, grey literature, and books, which may be based on primary data, were not included in the review. There is thus a possibility that these sources may have yielded additional results. Secondly, the inclusion criteria focused on Indigenous peoples of Australia and hence the health seeking behaviours of other groups of Indigenous people may not be reflected in this review. Lastly, since the review only included literature published in English (which was a practical consideration due to authors’ language limitations as well as to capture high-quality, peer-reviewed literature), the findings ignored the other language-based available evidence.

## Conclusion

Regular preventive health checks are an important component of current health care services. In Australia, the implementation of annual health checks for all Indigenous people over the age of 55 was instigated in 1999 and for over 15 years since 2004 (mainly the MBS item #715). This scoping review was undertaken to investigate the uptake/engagement of health checks/assessments by Indigenous people of Australia and to identify the barriers or enablers affecting Indigenous persons’ engagement and access to health assessment/health checks. The results indicate that there have been improving rates of uptake of health assessments by some sections of Indigenous communities. Our study found that Indigenous men and women living in rural areas need special attention in relation to regular health uptake. Aboriginal Australians are often found reluctant to uptake health assessments due to shame, recurring time needed for treatment purposes, culturally sensitive/intrusive questions associated with treatment procedures, and lack of health services. This study strongly suggests the need to ensure quality and culturally appropriate health services and Indigenous health leadership to improve the uptake of routine health assessments by Indigenous poeple.

It is also necessary for the Australian government to introduce new policies to support and encourage the regular uptake of health assessment by Indigenous People of Australia and provide the resources and services to ensure this occurs.

## Data availability statement

The original contributions presented in the study are included in the article/Supplementary material, further inquiries can be directed to the corresponding author.

## Author contributions

KU conceived the idea. KU and RJ developed the protocol. RJ and HK undertook the literature search and data extraction. KU, RJ, HK, and DJ conducted the data analysis. All authors contributed to the article and approved the submitted version.

## Conflict of interest

The authors declare that the research was conducted in the absence of any commercial or financial relationships that could be construed as a potential conflict of interest.

## Publisher’s note

All claims expressed in this article are solely those of the authors and do not necessarily represent those of their affiliated organizations, or those of the publisher, the editors and the reviewers. Any product that may be evaluated in this article, or claim that may be made by its manufacturer, is not guaranteed or endorsed by the publisher.
