# Supplementary material for: Preventative health assessments and indigenous people of Australia: a scoping review
Source: Front Public Health. 2023 Sep 6;11:1168568. doi: 10.3389/fpubh.2023.1168568 (PMC10509761; doi:10.3389/fpubh.2023.1168568)
Supplement: Supplementary file 1 [file Table_1.DOCX]

**Supplementary findings& illustration table**

| Citation | Findings/illustration | Narrative synthesis  Theme |  |  |
| --- | --- | --- | --- | --- |
| Bailie et al. (4) | **Findings**  Aggregated data show a general improvement in uptake of health assessments and follow-up items after the baseline period  Illustration  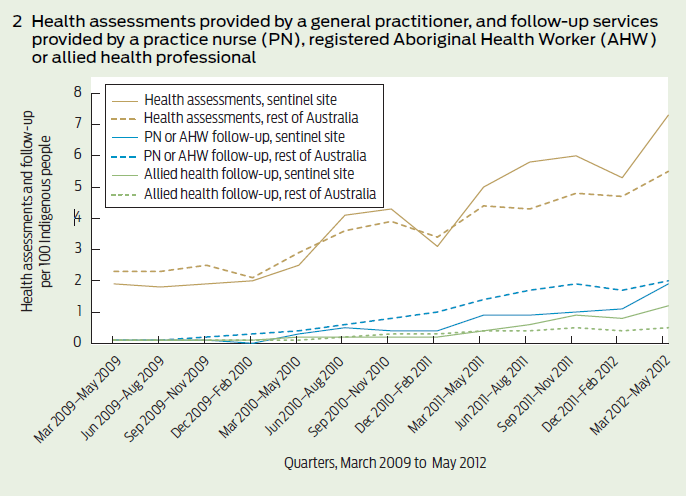 | uptake/engagement |  |  |
|  | **Findings**  Barriers and enablers to delivery and billing of follow-up care using a socioecological framework were identified at five levels of influence: patient, interpersonal, health service, community and policy.  **Illustration** |  | Not specific to HA rather specific to follow-up care so probably can’t use these. |  |
|  | **Negative past experiences affected patients’ willingness to attend follow-up appointments.** |  |  |  |
|  | **-Health service providers felt that short consultation times meant they had limited opportunity to explain reasons for referral for follow-up care to patients. This was related in part to shortage of service providers, including GPs, allied health professionals, Aboriginal Health Workers (AHWs) and practice nurses** |  |  |  |
|  | **Barriers related to Indigenous social and economic disadvantage included poor availability of transport to attend follow-up appointments and high or unpredictable cost of allied health services.** |  |  |  |
| Butler et al. (14) | **Findings**  Approximately one-third of participants received a Medicare-funded health check over a 2-year period in this large population-based study of Aboriginal and Torres Strait Islander adults living in NSW.  **Illustration**  Overall, 32% of participants had received at least one health check in the 2-year period from 1 January 2014 to 31 December 2015 | uptake/engagement |  |  |
|  | **Findings**  More women than me had a health check, and more like to have a health check lived regional/remote and inner regional compared to inner city.  **illustration**  Female 35.4% & Men 27.9% received 1 health check. The probability of receiving a health check was higher for women than men (adjusted OR 1.47; 95% CI 1.18, 1.84), for those with lowest versus highest education (OR 1.58; 95% CI 1.11, 2.24), and for those living in inner regional areas (OR 1.56; 95% CI 1.22, 2.01) or outer regional/remote areas (OR 2.38; 95% CI 1.8, 3.16) versus major cities.  Major city 24.7%, inner regional 33.3% & Outer regional/remote 4.7% | predictors of uptake/engagement |  |  |
|  | **Finding**  Use of GP services and poorer self-rated health remained strongly associated with receiving a health check  **Illustration**  Self-rated health  excellent 18.7%  Very good/excellent 21.9%  good 33.7%  fair 45.1%  poor 46.6%  missing 33.3%  GP Use  0-2 visits/yr 4.5% (  3-10 visit/yr 22.1%  ≥10 visits/yr 48.3% | predictors of uptake/engagement |  |  |
| Dutton et al. (15) | **Findings**  Over 2 years total of 1169 health checks/assessments where attended at Orange Aboriginal medical services (OAMS)  **Illustration**  1169 AHAs were performed: 52% (612) in 2011 and 48% (557) in 2012 (Table 1). Of these, 148 had two AHAs, none had more. | uptake/engagement |  |  |
|  | **Findings:**  **Risk factors where identified at the HA, The most common risk factor identified was being overweight. smoking,**  **Illustration**  At least one risk factor or morbidity was newly identified in 84% (984) of cases**.** Risk factors increased with age,  with 93% of older persons (67), 87% of adults (533) and 24% of children (117) with at least one risk factor.  **37% (178)**  received at least one intervention: dietary advice (26%; 122), physical activity (13%; 63), general weight loss (9%; 44); referral to an exercise physiologist (7%; 32), dietitian (4%; 17), healthy  lifestyle program (<1%; 3); and/or healthy lifestyle review (3%; 13).  Smoking was also a common risk factor, with 46% (285) of adults and19%(14) of older persons currently smoking. Of the 50 cases who intended to quit now, 60% (30) received at  least one intervention: 10 received smoking cessation advice, 10 were prescribed anti-smoking medication and 22 were referred to a smoking cessation program, of which 11 attended. | benefits |  |  |
|  | **Finding**  New diagnosis occurred during the HS; with skin, ear and dental problems the most common  **Illustration**  Minor skin and ear problems were the most common (12% (136) and 5% (57), respectively), especially in children.  Dental problems requiring referral were common among all AHA types (52%; 605). Of the 51% (599) of cases that received a referral, 50% (301) attended at least one appointment. An  additional 10% (126) of cases without an identified dental problem received a dental referral. | benefits |  |  |
|  | **Findings**  most common management that occurred due to findings in HA included advice, vaccination, and referral  **Illustration**  Overall, 41% (475) of cases received advice; 27% (311) were prescribed new medication; 13% (147) were vaccinated;  41% (479) had at least one blood test ordered and 32% (379) had further investigation;  70% were given at least one referral, most commonly to a dentist; and 42% (492) were advised to return for review.  Across all age groups, the most common advice given was for diet, physical activity or oral hygiene. | benefits |  |  |
| Jennings et al. (19) | **Findings:**  The AMS lacked a service-wide approach for conducting health checks (HCs), with different systems between clinics, and different systems recounted by staff within clinics. Although staff considered that a system-wide approach to HCs would facilitate increased uptake, they also felt strong local leadership and good communication was needed to help develop clinic-specific systems to embed the HCs as routine practice within busy workplaces.  **Illustrations:**  it needs like a practice manager who’s there to make sure it’s functioning and without that it’s quite ad hoc . . . and so doing something extra like a health check just becomes sort of an extra burden rather than a routine practice (Dr H18)  ‘. . . that no one’s got together and we don’t have a system’ (RN H20). | barrier | Health workers perspective  including A/TSI workers/nationality |  |
|  | **Findings**:  The busyness of the clinics and the length of HCs were frequently mentioned as barriers, This time barrier was compounded for clients with complex health care needs, or when consultations were preceded by a lengthy period in the waiting room. Staff considered that the ideal health check timeframe was ~30 min with the AHWs followed by ~30 min with the doctor.  **Illustration**  ‘. . . it’s like the size of War and Peace!’ (AHW H16A)  ‘. . . with the Indigenous people. . .you don’t keep them for a long time. . .otherwise they’ll just get up and go out. . .’(RN H01-aboriginal). | barrier |  |  |
|  | **Findings**  others relied heavily on walk-in consultations and opportunistic HCs because planned long appointments were often poorly attended.  **Illustration**  . . . in order to get a health check here you have to have an appointment, so . . . that’s one of the biggest barriers . . . you know ‘oh, come back next week for a health check, yeah?’ no, they’re not going to come back . . . they got what they need now . . . we really need opportunistic health checks . . . that’s what we need . . . (AHW H15) | barrier |  |  |
|  | **Findings:**  The lack of a clear system for conducting HCs, including a lack of clarity about the specific role of AHWs, doctors and nurses with the HCs was discussed by each group of staff. Some clinics expected AHWs to initiate the HCs, but AHWs had mixed levels of confidence with this role and some felt the doctor had more authority to initiate the HC, especially with difficult clients.  **Illustration**  Well the doctor would always let me know if they wanted that health check done. Yeah the doctor’s got to talk to the patient first and then get back to the health worker. (AHWH10b) | barrier |  |  |
|  | **Findings**  All staff were positive about the utility of the HC, with doctors focussing on its usefulness in diagnosing and managing complicated patients, while AHWs focussed on benefits of prevention and early identification of chronic disease. The financial benefits to the clinic and to clients were also acknowledged, including subsidised medications and allied health consultations.  **Illustration**  if we can start actively promoting this, we can possibly get better health outcomes for the younger generation in the future, so instead of coming to us with a chronic disease like our middle age people are, our younger people are coming to us with no chronic diseases. ’Cos it goes to the generations. (AHW H15) | benefits |  |  |
|  | **Findings**  Staff from all groups identified sections of the HC they perceived as difficult, sensitive or invasive. These included lifestyle factors, particularly alcohol and smoking, but also the social history, including current home environment and overcrowding. There was also concern about limited referral and follow-up capacity.  **Illustration**  I felt like it was [Department of Communities] you know, the department, asking some of those questions, ‘how many people living in your house?’. . . that’s not too bad, it’s starting to get a little bit invasive but, ‘does the mother drink, does the father drink?’, ‘how much do they drink?’. . . What’re we trying to achieve? We know we’re gonna get social issues with a lot of these kids. So once you find out that Dad drinks every, whatever, or they’re all smoking in the house, then what do you do? Again it comes back to capacity around implementing that information . . . (RN H07 Aboriginal)  . . . I think the hardest thing about the health check probably for both parties would be the lifestyle stuff, because that’s the most personal . . .’ (Dr H18). | barrier |  |  |
|  | **Findings**  Poor client engagement was identified, with difficulties getting parents to attend with children for HCs at the Murri school clinic an example.  **Illustration**  when you have some capacity-building and some understanding of the importance of having these health checks done regularly by the parents . . . if you haven’t got that level of engagement, it’s really an uphill battle. (RN H07 Aboriginal) | barrier |  |  |
|  | **Finding**  All Aboriginal and Torres Strait Islander staff but one, explicitly and without prompting, identified community health promotion and outreach as important enablers to improve HC uptake  **Illustration**  You know, we need to advertise it . . . we need client[s] to approach us and . . . request it . . . both parties have to agree and . . . like want it from the heart. We need to educate our people more. Tell them about the health check, the importance of health check. We need posters and pamphlets or whatever we can to provide that information to our clients, ’cause I look at here . . . it’s like 20 years back [than at home] . . . like in the health knowledge of the community, like in the conscious[ness].’ (AHW H13) | enablers |  |  |
| McAullay et al. (16) | **Findings**  Audit on patient medical records reported an increase in child health checks recorded in medical records  **Illustration**  significantly improved from 84% (n = 357) in 2011 to 95% (n = 415) in 2013 (OR 2.44, 95% CI 1.44–4.11) | uptake/engagement | medical record audit- subjective  focus is on recording of data related to health checks  health checks for children not adults  lacking indigenous voices |  |
|  | **Findings:**  All other child health check items showed statistically significant improvements over time (skin, oral, ears, hearing, development, interaction)  **Illustration:**  Weight checks remained consistently high (96–98%), and haemoglobin checks remained low (52–66%) from 2008 to 2013.  Hearing assessment improved the most, from 52% (n = 105) in 2008 to 89% (n = 378) in 2013 (OR 2.17, 95% CI 1.60–2.94). Skin checks improved from 73% (n = 309) | uptake/engagement  or ? improved reporting |  |  |
| Panaretto et al. (17) | **Findings**  Aboriginal and Torres strait islander Attendance at clinics increased  **Illustration**  from 27,369 2010 to 55,441 in 2012 | uptake/engagement |  |  |
|  | **Findings;**  The aggregated performance of participating services  for health assessment increased over time (figure 2). In October 2011, 8697 (44.1%—43.4, 44.8) of the regular patients had a current health assessment.  **Illustration**  **see image** | uptake/engagement |  |  |
| Reid et al. (20) | **Finding**  All caregivers reported the Share and Care Check was culturally appropriate, and the majority also reported that it was helpful  **Illustration**:  (n = 23; 85.2%). | benefits | quant data from indigenous voices |  |
|  | Finding:  A key positive feature noted by caregivers was the comprehensive nature of the health check.  **Illustration**  (n = 11; 40.7%) | benefits |  |  |
|  | **Finding**  four caregivers reported that the health check took too long.  **illustration**  (14%) | barrier |  |  |
| Robertson et al. (18) | **Findings:**  Our models demonstrate statistically significant reductions in total First Nations people health assessments during March, April, and May 2020. This data correlate with containment measure timelines and provides an early snapshot of the impact of such measures on First Nations people health assessments during the pandemic.  **Illustration**:  There was no significant difference between observed and predicted First Nations people health assessments in January, February, and June 2020. statistically significant decrease in health assessments in March (16.5%), April (23.1%), and May 2020 (17.2%) 2020. (National containment measured imposed in march) | uptake/engagement | quantitative data only |  |
|  | **Findings**  Telehealth health assessments did not entirely mitigate the reduction in face-to-face health assessments for First Nations people during the first wave of the COVID-19 pandemic (National containment measured imposed in march)  **Illustration**:  The proportion of total health assessments delivered via telehealth was 0.5%, 23.6%, 17.6%, and 10.0% for March, April, May, and June 2020, respectively | uptake/engagement |  |  |
| Schütze et al. (3) | **Finding**  positives to use HA & MBS-715  the MBS-715 allowed for earlier chronic disease detection and intervention  it remunerated GPs for the additional time spent with Aboriginal and Torres Strait Islander patients | benefits/enabler | health professional voices  no quotes used to support    **Lacking in the Indigenous voices** |  |
|  | **Findings**  Negatives to use HA & MBS-715  the MBS item number system was complicated and laborious, and billing any health assessment was avoided as a result or because of the work involved to try and recoup money if Medicare claims were rejected because patients has already had an MBS-715 billed elsewhere.  nursing staff at their practice were not actively involved in health assessments, which GPs considered too time consuming to undertake without this support. | barrier |  |  |
| Spurling et al. (21) | **Finding**:  Most key informants had had a HA with only two saying they had never had one. Key informants’ experience of Aboriginal and Torres Strait Islander HAs were mixed, as four key informants gave unqualified support for the capacity of Has to detect medical problems early.  **Illustration**:  I think the health checks are really important for Aboriginal and Torres Strait Islander people because some people . . .feel shame to go to the doctor, and if they leave it too long there could be a problem building in their body [Liam]. | HA limitations/barrier | Indigenous voices |  |
|  | **Findings**  In the context of a discussion about the effect of confidence and SCEDH, Edward felt that HA content was superficial and did not get to the heart of peoples’ health problems:  **Illustration**:  I’m not sure whether it paints a really honest picture of exactly where my health’s at. I think that [it] probably can go a bit more in depth [Edward]. | HA limitations |  |  |
|  | **Findings**:  Participants’ responses suggested that their view of health and the social world was not adequately covered by HAs, which measured health in a compartmentalised, disease-focussed way. For example, Bradley felt doctors and HAs were unlikely to help with his past concerns about identity, a social issue central to his health:  **Illustration**  I don’t see how a doctor is going to solve an identity crisis. It’s a social thing. . . [Bradley]. | HA limitations |  |  |
